# Construction of a potato consensus map and QTL meta-analysis offer new insights into the genetic architecture of late blight resistance and plant maturity traits

**DOI:** 10.1186/1471-2229-11-16

**Published:** 2011-01-19

**Authors:** Sarah Danan, Jean-Baptiste Veyrieras, Véronique Lefebvre

**Affiliations:** 1 Institut National de la Recherche Agronomique (INRA), UR 1052 Génétique et Amélioration des Fruits et Légumes (GAFL), BP94, 84140 Montfavet, France; 2 Institut National de la Recherche Agronomique (INRA-UPS-INA PG-CNRS), UMR 320 Génétique Végétale, Ferme du Moulon, 91190 Gif-sur-Yvette, France

## Abstract

**Background:**

Integrating QTL results from independent experiments performed on related species helps to survey the genetic diversity of loci/alleles underlying complex traits, and to highlight potential targets for breeding or QTL cloning. Potato (*Solanum tuberosum *L.) late blight resistance has been thoroughly studied, generating mapping data for many Rpi-genes (R-genes to *Phytophthora infestans*) and QTLs (quantitative trait loci). Moreover, late blight resistance was often associated with plant maturity. To get insight into the genomic organization of late blight resistance loci as compared to maturity QTLs, a QTL meta-analysis was performed for both traits.

**Results:**

Nineteen QTL publications for late blight resistance were considered, seven of them reported maturity QTLs. Twenty-one QTL maps and eight reference maps were compiled to construct a 2,141-marker consensus map on which QTLs were projected and clustered into meta-QTLs. The whole-genome QTL meta-analysis reduced by six-fold late blight resistance QTLs (by clustering 144 QTLs into 24 meta-QTLs), by *ca*. five-fold maturity QTLs (by clustering 42 QTLs into eight meta-QTLs), and by *ca*. two-fold QTL confidence interval mean. Late blight resistance meta-QTLs were observed on every chromosome and maturity meta-QTLs on only six chromosomes.

**Conclusions:**

Meta-analysis helped to refine the genomic regions of interest frequently described, and provided the closest flanking markers. Meta-QTLs of late blight resistance and maturity juxtaposed along chromosomes IV, V and VIII, and overlapped on chromosomes VI and XI. The distribution of late blight resistance meta-QTLs is significantly independent from those of Rpi-genes, resistance gene analogs and defence-related loci. The anchorage of meta-QTLs to the potato genome sequence, recently publicly released, will especially improve the candidate gene selection to determine the genes underlying meta-QTLs. All mapping data are available from the Sol Genomics Network (SGN) database.

## Background

The number of publications reporting the mapping of QTLs (quantitative trait locus) in plants has exponentially increased since the Eighties, reaching a total of about 34,300 papers in 2010 (source: Google Scholar with key words "QTL" and "plant"). For a few species only, this huge amount of QTL data has been recorded in databases that enable quick comparison of QTL mapping results from independent experiments (e.g. Gramene for maize and rice). But for most species, QTL data accumulates in bibliography until the coming out of hot-spot genomic regions that become targets for introgression into breeding material or for cloning. To get a comprehensive understanding of the genetic control of a polygenic trait and to optimize its use in breeding, it is needed to get a complete view of the genetic architecture of the trait with the distribution of the involved loci along the genome. This synthesis can be greatly facilitated by achieving a QTL meta-analysis.

The general principle of a meta-analysis is to pool the results of several studies that address the same issue to improve the estimate of targeted parameters. Meta-analysis was first used in social and medical sciences, like epidemiology. More recently, it was applied in plant genetics to combine on a single map the genetic marker data and the QTL characteristics (location, confidence interval, effect and trait used for QTL detection) from independent QTL mapping experiments to finally estimate the optimal set of distinct consensus QTLs, called meta-QTLs. The positions of those meta-QTLs are estimated with a higher accuracy as compared to the individual QTLs in the original experiments [1]. To date, QTL meta-analyses have been achieved for traits related to plant development and plant response to environment (nutrients, abiotic and biotic stresses) in maize, wheat, rice, rapeseed, cotton, soybean, cocoa and apricot [2-18].

Statistical methods have been proposed for the meta-analysis of QTLs from several experiments. The method proposed by Goffinet and Gerber (2000) was implemented in the Biomercator software [1,19]. It compiles the QTLs that have been projected on an existing reference map and uses the transformed Akaike classification criterion to determine the best model between one QTL, two QTLs, three QTLs etc. until the maximum number of QTLs mapped in the same region. This method was first used by Chardon *et al*. (2004) and by most authors until recently [2,3,6,8-10,15,16]. Then Veyrieras *et al*. (2007) have extended the statistical method and implemented the new algorithms in the MetaQTL software [20]. MetaQTL notably uses a weighted least squares strategy to build the consensus map from the maps of individual studies and offers a new clustering approach based on a Gaussian mixture model to define the optimal number of QTL clusters or meta-QTLs on each chromosome that best explain the observed distribution of the individual projected QTLs. The Gaussian mixture model has shown to be flexible and robust to the non-independence of the experiments [4]. Moreover, simulations demonstrated that the number of meta-QTLs selected by the Akaike criterion is lower than the expected number with random distributions of QTLs and that it has a very low probability to happen by chance [4]. The MetaQTL software has successfully been used in wheat, maize, rice and apricot [4,5,12,13,17].

Potato (*Solanum tuberosum *L.) late blight resistance is typically a trait for which meta-analysis can be applied. From 1994 to 2009, 19 studies have been published on QTL mapping in different crosses and with different related species, generating a significant amount of QTL data. All these publications reflect the interest of the potato scientific community towards polygenic partial resistance to late blight. Late blight, caused by the oomycete *Phytophthora infestans*, is one of the most serious diseases in potato, which is the third most important food crop in the world after rice and wheat. Almost all Rpi-genes (R-genes to *P. infestans*) deployed in the potato fields have been rapidly overcome, while polygenic resistance appears to be a fairly efficient and durable alternative. However, it has been observed that this kind of resistance in potato is often associated with plant maturity, as most resistant plants are also the ones that mature the latest. This is a handicap for breeders and growers who aim to get early maturing plants to shorten the time of tuber production.

Attempts to get a synthetic view of the loci controlling polygenic late blight resistance in potato with comparison of their positions with maturity QTLs have already been published [21,22]. However, because of a lack of common markers, the comparison of QTLs was achieved at a half-chromosome scale, which made the compilation imprecise. Consequently, to enhance the comparison of QTL positions coming from different mapping studies and also to refine the localization of hot-spot genomic regions, the mapping of common markers between maps is crucial.

Reference dense maps constructed with transferable markers are privileged sources of common markers. A UHD potato map containing 10,000 AFLP markers has been designed to become a reference map [23,24]. However, the anchorage of AFLP markers is restricted to closely-related species. In addition, as the comparison is based on the comigration of the marker bands on the gel, AFLP gels are required, which does not make the comparison easy to achieve [25]. Other reference maps containing SSR and RFLP markers have been developed in potato (SSR maps [26-28]; RFLP map [29]). These markers are well defined by specific primers or a probe sequence, which makes them easily transferable from one cross to another, even between distantly related species; they are thus handy tools for map comparison.

A functional map for pathogen resistance, enriched with RGA (resistance gene analog) and DRL (defence-related locus) sequences, SNPs and InDels tightly linked or located within NBS-LRR-like genes, has been developed on the basis of two potato populations (BC916^2 ^and F1840 [30-33]; PoMaMo database [34]). This functional map also contains CAPS, SSR and RFLP literature-derived markers, which enables the comparison with other QTL maps. However, it remains difficult to infer precisely functional locus information to QTL mapping results as QTLs often have large confidence intervals.

QTL meta-analysis thus appears here to be an adequate tool *i*) to narrow-down the confidence intervals of hot-spot loci where congruent late blight resistance QTLs of multiple origins map, and *ii*) to investigate colocalization of these loci with Rpi-genes, RGAs, DRLs and maturity QTLs as well. In this paper, we present a three-step meta-analysis process achieved with the MetaQTL software. First, we built a consensus potato map by compiling 21 QTL maps and eight reference maps. This consensus map includes common markers and specific markers tagging Rpi-genes, as well as RGA and DRL markers. Second, individual QTLs for late blight resistance and maturity were projected onto the consensus map. Third, for each trait, QTLs were clustered into meta-QTLs on the basis of the distribution of their projected positions on the consensus potato map.

## Results

### Bibliographic review of QTL mapping studies

The initial map set comprised a total of 37 maps divided into *i*) 29 QTL maps from 19 publications related to QTL detection of late blight resistance and maturity type, and *ii*) eight independent reference maps (without any QTL) (Table 1). Reference maps were included because they provided numerous pivotal markers, which improved connections between maps. Because of a lack of shared markers, the initial 29 QTL map set was refined to a core subset of 21 "connected" QTL maps coming from 14 publications that were included in the meta-analysis (Table 1).

**Table 1:** Number of publications, maps and QTLs collected to perform meta-analysis

	No. of publications	No. of maps	No. of QTLs
Available published data	19 (7) ^†^	29 (8)	211 (64)
Data included in meta-analysis ^††^	14 (4)	21 (5) + 8 ^†††^	144 (42)

First number: for late blight resistance traits; second number within brackets: for maturity traits.

† Table 2 lists all the concerned publications.

†† Only QTL maps that had a minimum of two common markers with at least a chromosome of another map were included into the meta-analysis.

††† 8 reference potato maps without QTLs (listed in Table 3) were added to meta-analysis to increase connections between maps through common markers and to improve consensus map accuracy.

The 21 "connected" QTL maps were representative of the diversity of assessments for late blight resistance and maturity, the QTL detection methods and the sources of resistance (Table 2). Resistance tests were based on disease spread on foliage in the field (FF) or in the greenhouse (FG), sporulation or necrosis spots on *in vitro *detached leaflets or leaf discs (LT), necrosis progression on stems (ST) and disease damage on tuber slices (TS) or whole tubers (T% or WT) in controlled conditions. Maturity type was evaluated by the number of days before flowering or senescence (MT), plant height (PH) and plant vigour (PV). QTLs were detected with different statistical detection methods according to the number of available markers, the size of the progeny and the frequency distribution profile of the raw or transformed data (non-parametric statistical tests or ANOVA, Interval Mapping, Composite Interval Mapping or Multiple QTL Mapping with permutation tests). Most of the *P. infestans *isolates used for late blight resistance assessments were of A1 mating type and virulent towards the 11 *S. demissum *Rpi-genes. However, it was difficult to say whether some of the isolates used in the different studies were the same or not. As wild tuber-bearing relatives of potato have proven to be high-potential sources of resistance, most mapping populations derived from a cross between a dihaploid *S. tuberosum *clone (the susceptible parent) and a clone derived from a diploid relative (the resistant parent). Two mapping populations even derived from crosses between two potato relatives (without *S. tuberosum*, Table 2). The parental pedigrees were sometimes quite complex. Nevertheless, the marker order in all maps was well conserved and aligned with the *S. tuberosum *map [35,36]. If all known species of the parent pedigrees are taken into account, a total of 13 potato-related species were involved in the meta-analysis.

**Table 2:** Published potato QTL mapping studies included in the QTL meta-analysis

Reference	Cross	**Pop. size**^**a**^	**No. of maps considered**^**b**^	**Resistance assay**^**c**^	**Maturity trait**^**d**^	**QTL detection method**^**e**^
[39] Bormann *et al.*, 2004	-*S. tuberosum *Leyla x *S. tuberosum *Escort	84	1 c	FF	MT	LR
	-*S. tuberosum *Leyla x *S. tuberosum *Nikita	95				

[55] Bradshaw *et al.*, 2004	-*S. tuberosum *12601ab1 x *S. tuberosum *Stirling	200-226	/	FF, FG, T%	MT, PH	LR

[68] Bradshaw *et al.*, 2006	-HB193 = HB171 (*S. tuberosum *PDH247 x *S. phureja *DB226) x *S. phureja *DB226	87-120	/	FF, FG, T%	/	IM

[42] Collins *et al.*, 1999	-GDE = G87D2.4.1[(DH Flora x PI 458.388) x (DH Dani x PI 230468)] x I88.55.6 {[DH (Belle de Fontenay x Kathadin) x PI 238141]x [DH Jose x (PI 195304 x WRF 380)]} **†**	113	2	FF, TS	MT, PV	LR

[35] Costanzo *et al.*, 2005	-BD410 = BD142-1 (*S. phureja *x *S. stenotomum*) x BD172-1 (*S. phureja *x *S. stenotomum*)	132	1 c	FF	/	IM

[38] Danan *et al.*, 2009	-96D31 = *S. tuberosum *CasparH3 x *S. sparsipilum *PI 310984	93	4	FF, ST	/	CIM
	-96D32 = *S. tuberosum *RosaH1 x *S. spegazzinii *PI 208876	116				

[54] Ewing *et al.*, 2000	-BCT = M200-30 (S*. tuberosum *USW2230 x *S. berthaultii *PI 473331) x *S. tuberosum *HH1-9	146	1 c	FF	/	LR

[69] Ghislain *et al.*, 2001	-PD = *S. phureja *CHS-625 x *S. tuberosum *PS-3	92	2	FF	/	IM

[41] Leonards-Schippers *et al.*, 1994	-P49xP40 = H82.368/3 (P49) x H80.696/4 (P40) **††**	197	2	LT	/	LR

[70] Meyer *et al.*, 1998	-*S. tuberosum *12601ab1 x *S. tuberosum *Stirling	94	/	FF	/	LR

[71] Naess *et al.*, 2000	-1K6 = J101K6 (*S. bulbocastanum *x *S. tuberosum*)] x *S. tuberosum *Atlantic	64	1 c	FG	/	LR

[64] Oberhagemann *et al.*, 1999	-K31 = H80.577/1 x H80.576/16 **†††**	113	1 c (K31)	LT	MT, PV	LR
	-GDE = G87D2.4.1 [(DH Flora x PI 458.388) x (DH Dani x PI 230468)] x I88.55.6 {[DH (Belle de Fontenay x Kathadin) x PI 238141]x [DH Jose x (PI 195304 x WRF 380)]} **†**	109				

[72] Sandbrink *et al.*, 2000	-89-13 = *S. microdontum MCD167 x S. tuberosum SH 82-44-111*	67	1 (MCD167)	FF	/	IM
	-*89-14 = S. microdontum MCD167 x S. tuberosum SH 77-114-2988*	46				
	*-89-15 = S. microdontum MCD167 x S. tuberosum SH 82-59-223*	47				
	*-89-16 = S. microdontum MCD178 x S. tuberosum SH 82-44-111*	82				
	*-89-17 = S. microdontum MCD178 x S. tuberosum *SH 77-114-2988	67				
	*-89-18 = S. microdontum MCD178 x S. tuberosum *SH 82-59-223	58				

[40] Simko *et al.*, 2006	- BD410 = BD142-1 (*S. phureja *x *S. stenotomum*) x BD172-1 (*S. phureja *x *S. stenotomum*)	125	1 c	WT	MT	MQM

[57] Sliwka *et al.*, 2007	-98-21 = DG 83-1520 (P1) x DG 84-195 (P2) **††††**	156	2	LT, TS	MT	LR

[73] Sorensen *et al.*, 2006	-HGG = *S. tuberosum *89-0-08-21 x *S. vernei *3504	70	1 c (HGG)	FF	/	MQM
	-HGIHJS = *S. tuberosum *90-HAE-42 x *S. vernei *3504	107				

[36] Villamon *et al.*, 2005	-PCC1 = MP1-8 (*S. paucissectum *PI 473489-1 x *S. chromatophilum *PI 310991-1) x *S. chromatophilum *PI 310991-1	184	1 c	FF, FG	/	CIM

[56] Visker *et al.*, 2003	-CxE = USW5337.3 (*S. phureja *x *S. tuberosum*) x USW5337.3 (*S. vernei *× *S. tuberosum*)	67	/	FF	MT	MQM

[58] Visker *et al.*, 2005	-Progeny 5 SHxCE = *S. tuberosum *SH82-44-111 x CE51 (*S. phureja *x (*S. vernei *x *S. tuberosum*))	227	/	FF	MT	IM
	-Progeny 2 DHxI =*S. tuberosum *DH84-19-1659 x I88.55.6 ((*S. tuberosum *x *S. stenotomum*) x *S. tuberosum *x *S. stenotomum*)	201				

^**a **^Population size for mapping; numbers could vary according to the phenotypic assessments for late blight resistance and maturity traits.

^**b **^A single number indicates the number of parental maps included in meta-analysis, otherwise the parental map which has been included is given; c: consensus map;/: no map was included because of a lack of common markers.

^**c **^Resistance assay: **FF**: foliage test in field, **FG**: foliage test in glasshouse, **T%**: tuber test in percentage of the number of infected tubers, **WT**: whole tuber test by scoring the tuber damage, **TS**: tuber slice test, **LT**: leaf test, **ST**: stem test.

^**d **^Maturity trait: **MT**: maturity type (assessment based on visual classification of senescence of the foliage), **PH**: plant height, **PV**: plant vigour.

^**e**^**LR**: linear regression, **IM**: simple interval mapping, **CIM**: composite interval mapping, **MQM**: multiple QTL mapping.

**† **G87D2.4.1 pedigree includes *S. kurtzianum*, *S. vernei, S. tuberosum*, and *S. tarijense; *I88.55.6 pedigree includes *S. tuberosum *and *S. stenotomum *[64].

**†† **P40 pedigree includes *S. tuberosum *and *S. spegazzinii *[41]

**††† **Unknown pedigree [64].

**†††† **Parental clone pedigrees of 98-21 population include *S. tuberosum, S. chacoense, S. verrucosum, S. microdontum, S. gourlayi, S. yougasense *[57].

### Consensus potato map

**Figure 1:**
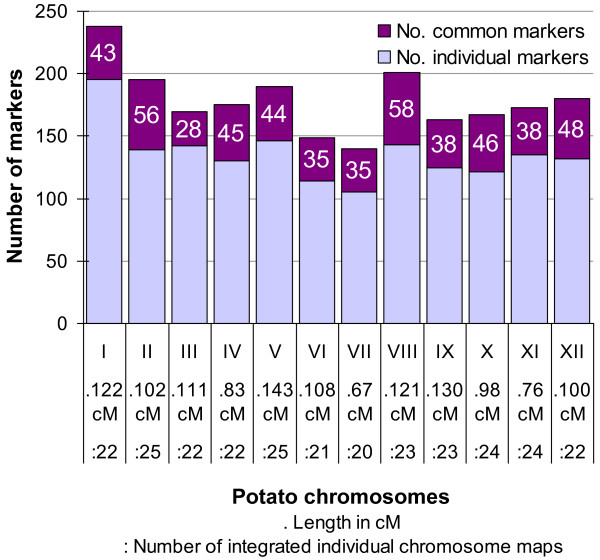
**Characteristics of the consensus potato map**. For each of the 12 potato chromosomes, the bar represents the total number of markers, the upper part corresponding to the proportion of common markers between at least two individual maps. The length of the consensus chromosome maps in cM (Haldane) and the number of individual maps used for their construction are indicated for each chromosome, below the bars.

Common markers between the 21 "connected" QTL maps and eight reference maps (Table 3) made it possible the construction of a consensus map for the 12 potato chromosomes. The number of maps used to construct each consensus chromosome varied between 20 and 25 (Figure 1). The consensus potato map had a total length of 1,260 cM (Haldane) and contained a total of 2,141 markers (SSR, SSCP, CAPS, RFLP, AFLP, SNP, InDels and STS markers). Among them, 514 markers were shared by at least two different maps. There were between 28 and 58 common markers per chromosome, corresponding to 16% up to 29% of the total number of markers per chromosome. The name, map position and occurrence of each marker are given in Additional file 1 and on the SGN database [37].

**Table 3:** Published potato reference maps included in the QTL meta-analysis

Reference	Cross	**Pop. size **^**a**^	**No. of maps considered **^**b**^	Marker types
[30,34] Gebhardt *et al.*, 1991 PoMaMo	-F1840 = H82.337/49 (P18) x H80.696/4 (P40) **††**	100	2 c	SSR, STS, RFLP, CAPS, BAC, pathogen resistance genes, DRL, RGA
	-BC916^2 ^= MPI= (H81.691/1 x H82.309/5) x H82.309/5)			

[28] Milbourne *et al.*, 1998	-Germicopa = GDE = G87D2.4.1[(DH Flora x PI 458.388) x (DH Dani x PI 230468)] x I88.55.6 {[DH (Belle de Fontenay x Kathadin) x PI 238141]x [DH Jose x (PI 195304 x WRF 380)]}	91	2 c	SSR, RFLP, AFLP, PCR-markers
	-MPI = BC916^2 ^= (H81.691/1 x H82.309/5) x H82.309/5)	67		

[26,29,37,74] Bonierbale *et al.*, 1988 Tanksley *et al.*, 1992 Feingold *et al.*, 2005 SGN	-BCB = N263 = M200-30 (*S. tuberosum *USW2230 x *S. berthaultii *PI 473331) x *S. berthaultii *PI 473331	150-155	2 c	SSR, RFLP
	-N271=BCT= M200-30 (S*. tuberosum *USW2230 x *S. berthaultii *PI 473331) x *S.tuberosum *HH1-9	150		

[27] Ghislain *et al.*, 2009	Integated SSR map based on SSR positions across 3 maps: BCT, PD, PCC1	92	1 c	SSR, RFLP

[75] Yamanaka *et al.*, 2005	*S. tuberosum *86.61.26 x *S. tuberosum *84.194.30	152	1 c	SSR, AFLP, CAPS

^**a**^**, **^**b**^**, ††**: detailed in the caption of Table 2.

### QTL dataset for meta-analysis

On the basis of the 19 publications of QTL studies, a total of 211 late blight resistance QTLs and 64 maturity QTLs were collected (Table 1). However, some QTL intervals did not include the minimum of two anchor markers, which were required for their projection onto the consensus map. Thus, the QTL dataset for meta-analysis was reduced down to 144 late blight resistance QTLs and 42 maturity QTLs, coming from 14 publications. The excluded QTLs, which harboured a single common marker with the consensus map, were referred to "anchored QTLs" and indicated at this marker position in Additional file 1 but their orientation and projected confidence interval could not be determined.

As far as the QTLs included in the meta-analysis are considered, late blight and maturity QTLs spread on the 12 potato chromosomes. The number of QTLs per chromosome ranged between six and 21 for late blight resistance, and between one and eight for maturity.

For late blight resistance, R² values were available for 106 QTLs out of the 144 input QTLs and ranged between 4% (chromosome I, foliage test [38]; chromosomes V, IX, XI, XII, foliage test [39]) and 63% (chromosome X, tuber test [40]). 75% of the late blight QTLs had relatively small effects, ranging between 4% and 15%; 7% of the QTLs had large effects, ranging between 30% and 63%. Confidence intervals ranged between 3 cM (chromosome III, leaf disc test [41]) and 66 cM (chromosome VI, foliage test [42]), with a mean of 24 cM.

For maturity, R² values were available for 20 QTLs out of the 42 input QTLs and ranged between 4% (chromosomes IX and XII [39]) and 71% (chromosome V [42]). 75% of the maturity QTLs had R² values ranging between 4% and 15%; 10% of the QTLs explained more than 30% of the phenotypic variation (60% and 71% on chromosome V [42]). Confidence intervals ranged between 4 cM (chromosome XI [42]) and 61 cM (chromosome VI [42]), with a mean of 20 cM.

### Meta-analysis

**Figure 2:**
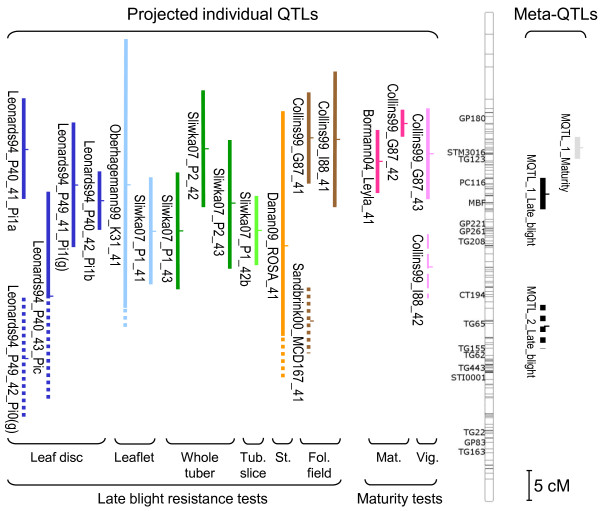
**Meta-analysis steps from QTL-projection on the consensus map to clustering into meta-QTLs: chromosome IV example**. Projected QTLs (quantitative trait loci) are represented by vertical bars to the left of the consensus chromosome IV. Their length is representative of their confidence interval once projected on the consensus map. They are sorted into assessment type, within late blight resistance traits (Leaf disc, Leaflet, Whole tuber, Tuber slice, Stem, Foliage in field), on one hand, and within maturity traits (Maturity, Vigour), on the other hand. QTL names are written to the left of the bars. QTL nomenclature is as follows: the name of the first author of the original publication juxtaposed to the last two digits of the publication year, the name of the population consensus map or of the parental map where the QTL was detected, and an Arabic number that can be followed by a letter. This latter Arabic number is the number of the chromosome juxtaposed to the QTL mapping order on the chromosome; a letter was sometimes added to distinguish colocalizing QTLs that were detected with different traits. For Leonards-Schippers *et al.*'s study, the original name of the QTL was added [41]. Ticks on the consensus chromosome indicate marker positions. Marker names are only shown for markers that occur at least in four maps out of the 21 compiled maps. Vertical thick bars to the right of the consensus chromosome indicate Meta-QTLs. Late blight meta-QTLs are in black and maturity meta-QTLs are in grey. Their length is representative of their confidence interval. To show clearly the results of the clustering step, the QTLs or part of the QTLs that were assigned to the 'MQTL_1_Late_blight' meta-QTL are in plain line and those assigned to the 'MQTL_2_Late_blight' meta-QTL are in dotted line. The QTL Collins99_I88_42 was not clustered to any late blight meta-QTL and was reported as an outlayer QTL in Additional file 1.

We determined the number of meta-QTLs per chromosome by using the modified Akaike Information Criterion (AICc) and by taking into account the consistency with the different criteria provided by the MetaQTL software (Additional file 2). Our analysis yielded a total of 32 meta-QTLs. Each meta-QTL corresponded to clusters of individual QTLs coming from different experiments. Meta-QTLs were composed of a maximum of 18 individual QTLs for late blight resistance (chromosome V) and eight individual QTLs for maturity (chromosome V). The QTL meta-analysis on the whole potato genome reduced by six-fold the initial number of late blight QTLs by passing from 144 QTLs to 24 meta-QTLs and by *ca. *five-fold the number of maturity QTLs by passing from 42 QTLs to eight meta-QTLs. Figure 2 presents an example of the meta-analysis steps for chromosome IV, from QTL projection on the consensus map to QTL clustering into meta-QTLs.

**Figure 3:**
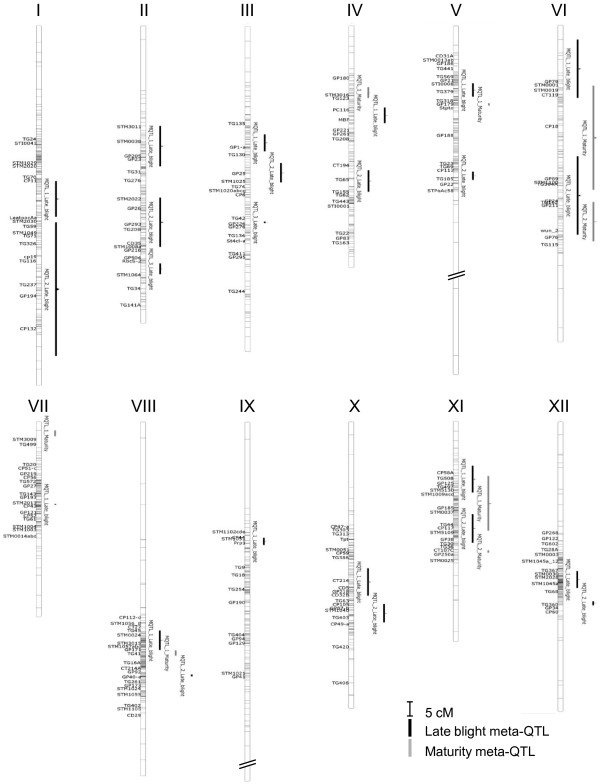
**Graphical overview of the late blight and maturity meta-QTLs**. The 12 consensus potato chromosomes are represented by 12 vertical thick bars. Ticks on the consensus chromosome indicate marker positions. Marker names are only shown for markers that occur at least in four maps out of the 21 compiled individual maps. Vertical thick bars to the right of the consensus chromosomes represent Meta-QTLs. Late blight meta-QTLs are in black and maturity meta-QTLs in grey. Their names start with "MQTL", followed by their position rank on the consensus chromosome from the top to the bottom of the chromosome, and the concerned trait used for clustering (Late_blight for late blight resistance trait and Maturity for maturity trait).

A graphical overview of the late blight and maturity meta-QTLs is presented on Figure 3. Late blight meta-QTLs spread on the 12 chromosomes, with one to three meta-QTLs per chromosome. Maturity meta-QTLs spread on only six chromosomes, with one or two meta-QTLs per chromosome. Other maturity QTLs were reported in literature on the other six chromosomes, but they were single in their region and no meta-QTL could be computed. Single QTLs for late blight resistance and for maturity that were excluded from the clustering step are shown in Additional file 1, with the other excluded QTLs which were anchored by a single marker to the consensus map.

The confidence intervals of late blight meta-QTLs ranged between 0.27 cM (chromosome VII) to 49.81 cM (chromosome I), with a mean of 10.25 cM (SD±10.79). The confidence intervals of maturity meta-QTLs ranged between 0.88 cM (chromosome V) to 39.28 cM (chromosome VI), with a mean of 10.67 cM (SD±12.54). With respect to the length reduction of the mean confidence interval from the published QTLs to the meta-QTLs, confidence intervals were reduced by 2.3-fold for late blight resistance and by 1.9-fold for maturity (Additional file 3).

Maturity meta-QTLs overlapped late blight meta-QTLs on chromosomes VI and XI, while there was no overlap on chromosomes IV, V, VII and VIII. However, by running meta-analysis on late blight resistance QTLs and maturity QTLs altogether under a single "super-trait", we observed that for all 12 chromosomes, maturity QTLs were always clustered together with late blight resistance QTLs in a "super meta-QTL" (data not shown). On the other way round, we observed at least one "super meta-QTL" free of maturity QTLs for 11 chromosomes; for chromosome XI only, both "super meta-QTLs" included at least one maturity QTL.

The three most consistent late blight meta-QTLs were located on chromosomes IV, V and X (MQTL_1_Late_blight of chromosome IV, MQTL_1_Late_blight of chromosome V and MQTL_2_Late_blight of chromosome X; Additional file 3). These meta-QTLs were composed of the highest number of QTLs (10 to 18 QTLs) with the largest effects (R² up to 63%, tuber test [40]). In addition, they corresponded to individual QTLs identified in different potato-related species or in plant material with complex pedigree. This result suggests that these regions could correspond to conserved resistance QTLs retrieved from several tuber-bearing *Solanum *species.

### Candidate gene analysis

Literature reported the map positions of several Rpi-genes determining late blight resistance (reviewed in [43,44]). However, only a few flanking markers were supplied (Rpi-genes were linked to a single marker or included in a large marker interval), which hampered the accurate location of Rpi-genes on the consensus map (Additional file 1). Due to their rough positions, it was thus not possible to say definitely whether they were included or not in the late blight meta-QTLs. Out of the 33 Rpi-genes positioned on our consensus potato map, 10 were included in the confidence intervals of late blight meta-QTLs (Table 4). One example of overlap was on chromosome IV, where the TG370-TG339 marker interval (~12 cM) containing a large NBS-LRR Rpi-gene cluster (*R2-like *genes) largely overlapped the meta-QTL MQTL_1_Late_blight [45]. On chromosome VI, the CT119 marker tagging the *Rpi-blb2 *R-gene was included in MQTL_1_Late_blight. On chromosome X, the TG422 and TG403 markers flanking the *Rpi-ber2 *gene were included in MQTL_2_Late_blight. However, on chromosome XI, the lack of anchor markers hindered the accurate location of the 10 Rpi-genes (*Rpi*-Stirling, *R5 *to *R11*, *R3a *and *R3b*). According to the flanking markers (STM5130-STM5109 for *Rpi*-Stirling, TG105-GP250 for *R3a*, TG26 for *R3b *and *R5 *to *R11*), only *Rpi*-Stirling might be included in MQTL_2_Late_blight.

**Table 4:** Number of collected individual QTLs, meta-QTLs, and colocalizations with Rpi-genes, RGAs and DRLs, per chromosome

**Chrom**.	No. maturity QTLs included in meta-analysis/No. QTLs	No. maturity meta-QTLs	No. late blight resistance QTLs included in meta-analysis/No. QTLs	No. late blight meta-QTLs	Rpi-genes positioned on the consensus map	No. Rpi-genes colocalizing with late blight meta-QTLs/No. Rpi-genes	No. RGAs colocalizing with late blight meta-QTLs/No. RGAs	No. DRLs colocalizing with late blight meta-QTLs/No. DRLs
I	3/3	0	10/10	2	-	0/0	0/10	4/8

II	1/1	0	6/7	3	-	0/0	5/7	5/8

III	3/4	0	15/21	3	-	0/0	0/2	0/5

IV	4/4	1	15/36**	2	*R2, R2-like, Rpi-blb3, Rpi-abpt, Rpi-demf1, Rpi-mcd, Rpi-mcd1*	7/7	2/7	1/5

V	8/29*	1	21/44***	2	*R1*	0/1	0/14	0/3

VI	5/5	2	8/12	2	*Rpi-blb2*	1/1	1/5	6/9

VII	3/3	1	9/12	1	*Rpi1=Rpi-pnt1*	0/1	0/4	0/2

VIII	6/6	1	12/13	2	*RB, Rpi-blb1, Rpi-pta1, Rpi-plt1, Rpi-sto1*	0/5	0/2	3/11

IX	1/1	0	9/13	1	*Rpi-vnt1.1, Rpi-vnt1.2, Rpi-vnt1.3, Rpi-phu1, Rpi-moc1= Rpi-mcq1*	0/5	0/2	1/9

X	1/1	0	15/18	2	*Rber*=*Rpi-ber1, Rpi-ber2*	1/2	1/8	3/6

XI	6/6	2	14/15	2	*R3a, R3b, R5, R6, R7, R8, R9, R10, R11, Rpi-pcs, Rpi-stirling*	1/11	5/18	3/3

XII	1/1	0	10/10	2	-	0/0	0/1	0/3

**Total**	**42/64**	**8**	**144/211**	**24**	-	**10/33**	**14/80**	**26/72**

* 18 QTLs, ** 15 QTLs, *** 17 QTLs, in Bradshaw *et al. *[55].

Conversely, a few Rpi-genes clearly did not belong to any late blight meta-QTLs. This was the case for *Rpi1 *on chromosome VII and for the *Rpi-vnt1*, *Rpi-phu1 *and *Rpi-mcq1/moc1 *loci of chromosome IX. In three additional cases, the distinction between Rpi-genes and late blight meta-QTLs was doubtful. On chromosome V, *R1 *gene (BA213c14 and BA87d17 BACs) was located less than 2 cM far below the lower bound of MQTL_1_Late_blight. On chromosome VIII, the *RB *cluster (*Rpi-blb1, Rpi-pta1, Rpi-plt1, Rpi-sto1*, tagged by RB marker) was located 1 cM far up to the upper bound of MQTL_2_Late_blight [46]. On chromosome X, the *Rber/Rpi-ber1 *locus was located between both meta-QTLs of this chromosome, in a 3-cM interval (Additional file 1).

In total, 80 RGAs and 72 DRLs were reported on our consensus map, mainly from the PoMaMo functional map [32,34]. Fourteen RGAs and 26 DRLs belonged to late blight meta-QTLs that covered about 20% of the consensus map (Table 4). Comparatively, 24 RGAs and nine DRLs belonged to maturity meta-QTLs that covered about 7% of the consensus map (Additional file 1). Independency Chi-2 tests indicate that the number of RGAs and Rpi-genes are under expectation in late blight meta-QTLs (p-value=0.035 under the hypothesis of independency) and over expectation in maturity meta-QTLs (p-value<0.0001). The heterogeneous distribution of RGAs and Rpi-genes corroborate the fact that they are often clustered or alleles. Conversely, the distribution of DRLs was independent on the distribution of both late blight meta-QTLs (p-value=0.323) and maturity meta-QTLs (p-value=0.909).

## Discussion

### A dense consensus reference potato map for map comparisons

Twenty-nine published potato maps were merged together into a single consensus map. From the information available in the publications of the genetic maps, at least three maps come from the cultivated potato species (*S. tuberosum*) and 23 maps from crosses between *S. tuberosum *and potato wild relatives (*S. microdontum*, *S. phureja*, *S. sparsipilum*, etc., Tables 2 and 3). Sixteen maps are already consensus maps of both parents, with generally one being a *S. tuberosum *clone and the other one a resistant wild potato species. This ability to compile genetic map information of *S. tuberosum *and its wild relatives indicates a high level of conservation of the marker order, and thus, of genomic sequences all over the genome. This stresses the very close genetic relationships of those genetic backgrounds and gives evidence of the validity to compile their deriving published QTL data produced with their maps. The genetic relationships between the cultivated potato and its wild relatives have been described in details by Spooner *et al*. (2008) [47].

Composed of 2,141 markers, the consensus map constructed in our study constitutes a new valuable dense reference map of potato. Marker positions are available on the SGN database, enabling map comparisons [37,48]. This map can be used either as a source of markers for regions of interest or as an anchor reference map. However, for regions with a high density of markers, precise marker order has to be taken with care as the marker positions were calculated according to the positions of common markers from different maps and not based on recombination fractions. Thus, precise marker positions have to be checked by mapping in a large population.

One feature of this consensus map is that markers are not regularly spread along the chromosomes and tend to concentrate in the medium regions. For example, on chromosome VIII, 18 markers are spread on the top 71 cM while 167 markers are spread on the next 29 cM. This phenomenon is indeed observed on the published maps where dense regions are often assimilated to centromeric regions characterized by a small number of recombinations and consequently compressed maps. We also assume that genomic regions known to be involved in late blight resistance mostly gather in medium regions, which had been enriched with markers. Another explanation would be that distal markers generally originate from a single published map and their positions could be due to genotyping errors or skewed segregations.

### A clear picture of the structural organization of late blight resistance loci on the potato genome

The synthetic potato map with meta-QTLs offers a refined overview of the structural organization of the loci of polygenic resistance to late blight in terms of number of QTLs and lengths of confidence intervals. By reducing the number of resistance loci by a factor of six (from 144 QTLs to 24 meta-QTLs), meta-analysis highlights the well-known resistance gene clusters on chromosomes IV and V, and also points out loci which had not appeared as notable in individual experiments like the loci on chromosomes I, III, VII, and XII. Twenty-four meta-QTLs summarized about 96% of the individual QTLs included in the analysis, which illustrates the power of QTL meta-analysis to combine QTLs from various studies.

One may question the validity of such QTL meta-analysis compiling information of as different species as the cultivated potato (*S. tuberosum*) and its wild relatives (*S. stenotomum*, *S. berthaultii*, *S. bulbocastanum*, etc.). However, as we explained earlier, this meta-analysis could only be performed thanks to the presence of common molecular markers mapped in a conserved order across maps of different related species. This structural tight genetic relationships of the different backgrounds sets up the hypothesis that the same genes are present in the same order in the genome across species, and that the genetic variation, if any, would take place at the allele level. The fact that the Potato Genome Sequencing Consortium is currently exploiting the high genomic similarity between *S. tuberosum *and *S. phureja *to reduce the complexity in assembly supports this hypothesis [49]. A high level of sequence conservation was also observed at the nucleotide level of the coding sequence among six Solanaceae genera (potato, tomato, pepper, petunia, tobacco and *Nicotiana benthamiana*) [50]. Because the number of sequence matches among different Solanaceae EST libraries was inversely correlated with the phylogenetic distance, we assume that the tuber-bearing species are also very similar at the level of expressed genes. These hypotheses have been already proposed in other genera where meta-analysis was conducted across relatives (e.g. for cotton fiber [15] and for rice blast [2]). In potato, resistance QTLs from one population frequently mapped, as far as resolution allows, in close proximity to those described in other populations. At the gene level, high sequence homology of *Rpi *genes were described between potato relatives. Functional homologues of the *R2 *resistance gene to *P. infestans *located on potato chromosome IV were cloned by an allele mining approach in three related species, and recognized the same effector protein of *P. infestans *[45]. *Rpi *genes and their general functions are overall well conserved across potato related species, variation being in fact limited to differences at the base pair and allele function levels [51]. At last, in a recent published study, meta-analysis was performed on populations involving different species related to bread wheat to narrow-down the interval of a QTL controlling the nitrogen use efficiency [52]. The functional underlying gene has been identified and showed to be conserved at orthologous positions in wheat species and in much further related cereals species such as rice, sorghum and maize. These different elements demonstrate that the analysis of the genetic factors controlling a trait across genomes of different related species and even of different genera of a plant family can be very powerful to perform a map-based dissection of a conserved gene that controls the same trait in several species.

In our study, the locus confidence intervals have been reduced by 2.3-fold in average. Locus accuracy has especially increased for the loci for which colocalizing QTLs are numerous, like on chromosome V where 18 colocalizing QTLs with an averaged confidence interval of 23 cM were clustered into a single meta-QTL of only 5 cM. In this way, meta-analysis refines the genomic regions of interest frequently described. This enables the determination of a set of markers for selection and a reasonable list of candidate genes when the genetic map is anchored to the annotated genome sequence. The closest flanking markers of the locus are also provided for subsequent fine-mapping in a real large population for map-based gene cloning.

However, confidence intervals of meta-QTLs have to be taken with caution, as they are the result of two successive statistical transformations (projection onto a consensus map and clustering), based themselves on the individual QTL confidence intervals. This stresses the importance of the accuracy of the initial mapping data. For our analysis, we took into account the individual QTL confidence intervals as described in the original publication when available (interval length of a certain LOD decrease). Otherwise, the confidence interval estimate was calculated with the empirical formula of Darvasi and Soller (1997) whose accuracy depends on the population size and the QTL effect [53]. To reduce the risk of giving artificially too much weight to a locus, we made a quite strict selection of the individual QTLs to be included in the meta-analysis by removing all possible redundancy (e.g. several repetitions or related traits in the same experiment). In our study, five meta-QTLs displayed confidence intervals lower than 1 cM (on chromosomes III, V, VII, VIII and XI), while the confidence interval of individual QTLs varied greatly. In these regions, the consensus map appeared condensed in comparison with the original maps; therefore, the projected confidence intervals of individual QTLs were very tight. Löffler *et al*. (2009) have also found in wheat a very tight meta-QTL of 0.1 cM that encompassed six QTLs only [12]. These over-reduced confidence intervals underline the necessity to validate the marker-trait association, either by association mapping or by transcriptomics when possible as performed by Norton *et al. *(2008) [14].

The meta-analysis implemented by the Meta-QTL software assumes that QTL experiments are independent from each other. Therefore, it could not take into account common features to several studies, such as the relatedness between mapping populations or between *Phytophthora *isolates that would have increased the power of the analysis. Nevertheless, by projecting QTLs on the same consensus map, meta-analysis still makes it possible to rapidly compare QTL mapping results of linked studies and highlights QTLs conferring isolate-specific resistance (same population and assessment but different isolates [54,55]) or with tissue-specificity effect (same population and isolate but assessments on foliage and tuber [35,40]). Another limit of the meta-analysis implemented in MetaQTL is that it does not provide the direction of the allelic effects, meaning that QTLs composing a meta-QTL can have opposite direction alleles. Consequently, we have to come back to the individual QTL data to be able to select the origin of the target favourable allele.

### Polygenic late blight resistance and maturity relationships

Late blight meta-QTLs overlapped maturity meta-QTLs on chromosomes VI and XI. In addition, if we consider individual maturity QTLs excluded from the meta-analysis but anchored with a single common marker, other overlaps were presumed with late blight meta-QTLs on chromosome I, II, V, and XII, and reciprocally individual late blight QTLs overlapped maturity meta-QTLs on chromosomes IV, V, VI, VII, and XI (Additional file 1). In these cases, either pleiotropic genes might control both traits, or the resolution is not accurate enough to distinguish two closely linked genes.

The most famous association between QTLs for late blight resistance and for maturity is located on the upper part of chromosome V [39,40,42,55-57]. Here, meta-analysis results show that the maturity meta-QTL MQTL_1_Maturity consisting in eight maturity QTLs reported for this chromosome was very close but distinct from the most consistent late blight meta-QTL MQTL_1_Late_blight consisting in 18 individual QTLs. This result goes rather in favour of the hypothesis that each trait would be controlled by independent genes but very closely linked. Nevertheless, this result has to be taken with care as two individual maturity QTLs that were not included in the meta-analysis were anchored to markers of the confidence interval of MQTL_1_Late_blight. Also, another individual late blight QTL was anchored to a marker of the MQTL_1_Maturity's confidence interval (Additional file 1).

Cases of clearly distinct maturity and late blight meta-QTLs were found on chromosomes IV, VII and VIII; the hypothesis of a pleiotropic gene would then be excluded for these regions. In addition, anchored individual maturity QTLs did not coincide with late blight meta-QTLs on chromosomes III and X, corroborating the independence of late blight and maturity loci in these regions as suggested in previous studies [39,40,55,58]. The clearest cases of physical independency between maturity and late blight resistance QTLs could be preferential targets for introgression into elite cultivars for late blight resistance breeding.

### Candidate genes for late blight resistance QTLs

Most frequent hypotheses about resistance QTLs were either defeated R-genes with residual effects or defence genes. The confidence intervals of the late blight meta-QTLs of chromosomes IV, VI, X and XI include R-genes of the NBS-LRR class (*R2 *cluster, *Rpi-blb2*, *Rpi-ber2 *and *Rpi-Stirling *respectively). The late blight resistance locus of chromosome IV was particularly well documented with the Stirling cultivar and *S. microdontum *cases of study [55,59,60]. The detection of the same locus as an R-gene or a QTL can be accounted for several factors such as the allelic form of the gene (for instance, defeated complete resistance alleles could be detected as QTLs), the composition of the pathogen isolate, the way of scoring the disease or the genetic background. Bhaskar *et al. *(2008) demonstrated that the resistance level conferred by the *RB *gene was dependent on the quantity of proteins encoded by the essential cell cycle regulator *SGT1 *gene. This showed the importance of the genetic background in the efficiency of R-gene-triggered disease resistance [61]. A parallel can be made with the fact that quantitative resistance is often controlled by a few large-effect QTLs in association with several minor-effect QTLs which can interact with the major QTLs to modulate the expression of the given trait (reviewed in [62]).

By analysing colocalizations between the 24 late blight meta-QTLs and the 33 Rpi-genes, 80 RGAs and 72 DRLs, we observed that 25% of late blight meta-QTLs included RGAs (33% included Rpi-genes or RGAs), and 50% included DRLs. It also appeared that the frequency of RGAs was not significantly greater inside late blight meta-QTL confidence intervals than outside, and that DRLs were neither significantly associated with late blight meta-QTLs, nor with maturity meta-QTLs. Even if our analysis was biased by the limited number of candidate genes and QTLs, our results do not favour one particular hypothesis for molecular basis of resistance QTLs rather than another, corroborating Ballini *et al*.'s conclusions [2]. However, the meta-analysis presents the advantage to reduce QTL confidence intervals, which contributes to increase the resolution in selecting relevant candidate genes. As an example, the *StAOS2 *gene encoding the potato allene oxide synthase 2 was located within the MQTL_2_Late_blight's confidence interval. Pajerowska-Mukhtar *et al. *(2008) showed that the natural variation of this gene was associated with a late blight resistance QTL identified by Oberhagemann *et al. *(1999) [63,64]. Such congruency between meta-analysis and fine mapping results was also reported for the *Vgt1 *QTL in maize [20,65].

## Conclusions

In our study, we produced the first consensus map and performed the first meta-analysis dealing with both development trait and resistance to a biotic stress in potato. Through this study, we demonstrated that, as soon as a large amount of QTL data is collected from different studies and connected by common genetic markers, meta-analysis becomes a powerful tool to highlight chromosomal regions to focus further researches on and to use in breeding. To narrow-down the target loci confidence intervals, it is thus worth systematically integrating all new QTLs into meta-analysis on a regular basis. The anchorage of the new annotated potato genome sequence to meta-QTLs will especially provide interesting targets for candidate gene approach and for marker-assisted breeding [66]. Meta-analysis could also be useful for comparative QTL mapping across widely related crops of the same family, as achieved between rice and maize [67]. This opens a new type of analysis that would integrate gene evolution and functional conservation.

To improve meta-analysis, it would be necessary to integrate the relationships between parental clones across experiments, along with their pedigree, to be able to determine the donors of resistant alleles. In addition, adding information on *P. infestans *isolates used for resistance assessments would enlighten on the resistance spectrum mediated by meta-QTLs, which is one of the predictors of the durability of resistance to pathogens. We assume that broad-spectrum meta-QTLs probably target essential functions of the pathogen and that meta-QTLs supported by QTLs detected from several genitors or related species probably provide a selective advantage. Consequently, we presume that meta-QTLs with a broad-spectrum and retrieved from different genitors correspond to constrained genes, and could therefore be preferential targets to increase the durability of the resistance.

## Methods

### Consensus potato map

The construction of the consensus map was performed chromosome by chromosome. To be able to align the chromosome maps in the right orientation, a chromosome of a study should contain a minimum of two common markers with the corresponding chromosome of another study. QTL maps that did not share common markers were discarded from the construction of the consensus map. For several maps, few chromosomes were also missing, which lead to a variation of the number of input maps depending on the chromosome (Figure 1).

In case of inversions of two markers between maps, only the marker that was present in the lowest number of maps was manually removed to ensure that the most frequent common markers would be systematically retained. We repeated this process until no more inversion was observed between maps.

The ConsMap command of the MetaQTL software version 1.0 was used to create the consensus marker map [20]. The implemented method is based on a Weighted Least Square (WLS) strategy, which made it possible to compile all the input maps into a consensus map in a single step. It takes into account the distances between adjacent markers from all individual maps rescaled in Haldane unit. The size and type of the mapping population are used to estimate the map accuracy and are integrated into the compilation. Marker names and positions were provided in the input map files, along with a file specifying the synonymous names of the same markers that were mapped in different maps (Additional file 4 and on the SGN database [37]).

### QTL meta-analysis

QTL meta-analysis was performed with the MetaQTL software version 1.0 [20]. MetaQTL requires a minimal set of descriptors characterizing each observed QTL: the QTL position, its confidence interval and/or its individual R² value (at least one of them is mandatory), the trait related to the QTL and the size of the QTL mapping population used for the QTL detection. The statistical method implemented in the MetaQTL software hypothesizes that the input mapping studies are independent from each other. QTL mapping studies, which were repeated in time and space, often detected redundant QTLs at the same position for the same trait. In that case, we kept only the QTL with the highest effect (R²) to avoid the attribution of a too strong weight to that QTL in the meta-analysis.

QTLProj command enabled the homothetic projection of the positions and the confidence intervals of the individual QTLs onto the consensus map. It is based on a scaling rule between the positions of the flanking markers of the QTLs on their original maps and the positions of these markers on the consensus map. The MetaQTL software first took into account the confidence interval reported in the study if available, otherwise an estimation of the confidence interval was calculated using the empirical formula proposed by Darvasi and Soller (1997) [53]: CI=530/NxR², where N is the population size and R² the QTL effect as reported in the individual study. This formula generally gives larger confidence interval than the usual interval length of LOD-1 decrease. We used trait ontology to classify and group original trait names according to their relatedness.

QTLClust command performed the clustering of the projected QTLs referring to the same trait on a given chromosome into all possible numbers of hypothetic clusters or "models", i.e., from the model consisting in only one hypothetic cluster to the model consisting in as many clusters as the total number of individual QTLs reported for the chromosome. For a given model, a Gaussian mixture approach was applied to jointly perform a quantitative clustering of the projected QTLs and estimate meta-QTL positions and confidence intervals by maximizing the likelihood of the initial QTL positions. The clustering could only be performed in the genomic regions where at least two QTLs overlapped. If QTLs were single in a genomic region (referred as outlayer QTLs in Additional file 1), they were excluded from the clustering step.

QTLModel command determined the best clustering model based on information-based criteria that were computed for each possible model: AIC (Akaike information criterion), AICc, AIC3, BIC (Bayesian information criterion) and AWE (average weight of evidence) [20]. The best model was the one which criteria values were the lowest relatively to the criteria values of the other possible models. It corresponds to the optimal number of clusters that best explain the observed QTL distribution along the consensus chromosome map. As a result, each meta-QTL position and confidence interval correspond to the consensus position of all the individual QTLs attributed to this meta-QTL, weighted by their individual accuracies and probability of being attributed to the meta-QTL.

A two-round QTL meta-analysis was adopted. First, a meta-analysis was performed by declaring late blight resistance distinct from maturity in the trait ontology; separated meta-QTLs were thus obtained for each trait in the same analysis. In the second round of QTL meta-analysis, both traits were merged into one single "super trait". The purpose of this second round was to investigate whether maturity QTLs tended to cluster with late blight resistance QTLs or not. For convenience, meta-QTLs of late blight resistance were called "late blight meta-QTLs", and meta-QTLs of maturity, vigour and plant height were called "maturity meta-QTLs".

## Authors' contributions

VL conceived of the initial idea and coordinated the study. All authors participated in the conception of the study. JBV originally coded the package MetaQTL and gave a significant support during the meta-analysis process. SD carried out the collection of data and applied the QTL meta-analysis. VL and SD contributed to the interpretation of the results and drafted the manuscript. All authors read and approved the final manuscript.

## Supplementary Material

Additional file 1 **Description of the consensus map with meta-QTLs positions, additional individual QTLs, Rpi-genes, RGAs and DRLs**. The chromosome Arabic numbers, the locus names, and the positions of the loci on the consensus map (in cM, Haldane unit) are listed in the ascending order of marker positions, from chromosome I to chromosome XII. The occurrence number of the markers across the 21 compiled maps is indicated (meta.occurrence). The "type" information indicates whether the locus is a marker (M) or a meta-QTL (Q). If the marker sequence has been previously described as being a resistance gene analog (RGA) or a defence-related locus (DRL) (information mainly retrieved from the PoMaMo database), the type "M_RGA" or "M_DRL" is mentioned; in this case, a short description about the sequence is added. Rpi-gene (R-gene to *Phytophthora infestans*) most probable locations are indicated by shaded areas between their two closest flanking markers, as described in literature. Unless markers were designed from known Rpi-gene sequences (as for *RB *gene), Rpi-genes have most of the time an approximate location on the consensus map. Overlapping locations of Rpi-genes in the same region are indicated in two different columns of the table. Rpi-genes with different names are considered as being different genes, even if they are alleles [44]. The columns "qtl.ci.from" and "qtl.ci.to" indicate the range of the meta-QTL confidence interval on the consensus map. In the table, the confidence intervals of late blight meta-QTLs are framed in blue while those of maturity meta-QTLs are framed in red, and overlapping regions in purple. The column "meta-QTL trait" specifies the meta-QTL trait: late blight resistance (Late_blight) or maturity (Maturity). The columns "anchored QTL, trait" and "outlayer QTL, trait" show additional QTLs that have not been compiled in the final meta-analysis. The QTL nomenclature is described in the Figure 2 legend. For "anchored QTLs", the final number is the chromosome number alone; for "outlayer QTLs", the final number is the chromosome number juxtaposed to the QTL mapping order on the chromosome and a letter was sometimes added to distinguish colocalizing QTLs that were detected with different traits. The trait with which the QTL was detected is indicated after the comma by the trait code, as defined in the Table 2 legend. "Anchored QTLs" are QTLs from non-compiled maps and they could only be anchored to the consensus map if the confidence interval comprised a common marker; they are mentioned at the position of the common marker. No confidence interval information is available. "Outlayer QTLs" are QTLs from compiled maps and, as they were alone in their region on the consensus map, they eventually could not be clustered with other QTLs in a meta-QTL. Confidence intervals of these "outlayer QTLs" are represented by dotted areas. Click here for file

Additional file 2 **Criterion values of the meta-QTL models**. The values of the AIC, AICc, AIC3, BIC and AWE criteria provided by the MetaQTL software are listed for the twelve chromosomes (first column), for both maturity and late blight resistance traits (second column), and for all possible meta-QTL models (third column, K is the number of hypothetic meta-QTLs of a model). "Delta" is the rescaled value of the criterion, it is the difference between the value of the given model and the value of the best model. The weight is the "weight of evidence" of the model, the higher it is, the more confidence we can have in the corresponding model. Click here for file

Additional file 3 **Meta-QTL details: number per chromosome, confidence intervals and composition in published QTLs**. For each chromosome, late blight and maturity meta-QTLs are described. Meta-QTL numbers, names and confidence intervals (CI, in cM, Haldane unit) are specified. The QTL composition of each meta-QTL is detailed by giving the individual QTL names, the species origins of the mapping parents (when pedigrees are known), R² values (if specified in the original publication) and confidence intervals as described in the original publication (in their original unit, Haldane or Kosambi) with their means for each meta-QTL. The confidence intervals of the projected QTLs with their means are also indicated. The QTL nomenclature is described in the Figure 2 legend. For Villamon *et al*. and Leonards-Schippers *et al*.' studies, the original name of the QTL was added [36,41]. When QTLs are equally shared by two meta-QTLs, they are shown in italic ('LB' for late blight resistance). The mean of the original confidence intervals of the QTLs and the number of the QTLs composing the meta-QTL are given for each meta-QTL. NA: not available. Click here for file

Additional file 4 **Synonymous names of markers described in the compiled published maps**. Marker names that were the most often encountered for a marker and which had a familiar nomenclature were considered as standard names. Their corresponding synonymous marker names that were found in the compiled maps were collected. Only standard names were used in the final consensus map. Click here for file
